# Stramonium in Neuralgic Affections

**Published:** 1841-09

**Authors:** 


### STRAMONIUM IN NEURALGIC AFFECTIONS.

Prof. Short has put into our hands a letter from Dr. J. W. Richardson of Rutherford county, Tennessee, in which he cites many facts in favor of stramonium, in different neuralgic disorders. He prepares the system of his patient by vomits, purges, venesections and low diet; and then gives half a grain of the extract of stramonium, prepared according to the U. S. Pharmacopœia, every six hours, till dimness of vision is produced. D.

